# A Bifactor Model of Subjective Well-Being at Personal, Community, and Country Levels: A Case With Three Latin-American Countries

**DOI:** 10.3389/fpsyg.2021.641641

**Published:** 2021-06-03

**Authors:** Javier Torres-Vallejos, Joel Juarros-Basterretxea, Juan Carlos Oyanedel, Masatoshi Sato

**Affiliations:** ^1^Facultad de Educación y Ciencias Sociales, Universidad Andres Bello, Santiago, Chile; ^2^Centro de Investigación para la Educación Inclusiva, Pontificia Universidad Católica de Valparaíso, Viña del Mar, Chile; ^3^Centro Universitario de la Defensa de Zaragoza, Zaragoza, Spain

**Keywords:** subjective well-being, country, community, bifactor model, measurement

## Abstract

Improving citizens' subjective well-being (SWB) has become an increasingly visible policy goal across industrialized countries. Although an increasing number of studies have investigated SWB at the individual level, little is known about subjective evaluation at social levels, such as the community and national levels. While the relationships between these levels have been analyzed in previous research, these assessments, which are part of the same unique construct of SWB, are under-investigated. The purpose of this study was to examine the dimensionality and reliability of a single measure of SWB, which contained individual, community, and national levels across three Latin-American countries (Argentina, Chile, and Venezuela), using a bifactor model analysis. Findings showed that the bifactor model exhibited a good fit to the data for the three countries. However, invariance testing between countries was not fully supported because of each item's specific contribution to both specific and general constructs. The analyses of each country showed that the SWB construct was in a gray area between unidimensionality and multidimensionality; some factors contributed more to the general factor and others to the specific level, depending on the country. These findings call for integrating more distant levels (community and country levels) into the understanding of SWB at the individual level, as they contribute not only to an overall construct, but they make unique contributions to SWB, which must be considered in public policy making.

## Introduction

Well-being has become a core concern in contemporary societies and has become an important issue in public policy across the globe (Easterlin, 2013; Frijters et al., 2020). In this regard, well-being is considered a good reflection of a given society's development and thus an appropriate way to evaluate the society (Diener et al., 2009); well-being has been showed to be related to several socially desirable outcomes, such as lower prevalence of mental illness (Lyubomirsky et al., 2005; Pressman and Cohen, 2005; Howell et al., 2007; Davidson et al., 2010; Kushlev et al., 2020), better mental health (Keyes, 2006; Sin and Lyubomirsky, 2009; Werner-Seidler et al., 2013; Germani et al., 2020), higher life expectancy (Diener and Chan, 2011; Zaninotto and Steptoe, 2019; Potter et al., 2020), higher educational attainment (Nickerson et al., 2011; Bücker et al., 2018), increased creativity (Dolan and Metcalfe, 2012), higher work productivity (Oishi, 2012; Bryson et al., 2017), a tendency toward prosocial behavior (Aknin et al., 2011; Helliwell et al., 2018; Su et al., 2019), and predictive capabilities toward depressive states and skills to deal with stressful life events (Lucas, 2007; Luhmann et al., 2012).

The increasing relevance of well-being to the public interest and public policy is reflected in the increase in research (Diener, 2013), with the increase of both theoretical and methodological proposals. The majority of empirical and theoretical advancement has been focused on the subjective well-being (SWB), which pertains to people's emotional and cognitive evaluations, both positive and negative, of how they perceive their own lives (Ryan and Deci, 2001; Diener et al., 2003; Wills, 2009). SWB is composed of (a) an emotional component, which includes experienced positive and negative emotions (Davern et al., 2007), and (b) a cognitive component known as satisfaction with life (Diener and Suh, 1997; Pavot and Diener, 2008), considered a global individuals' assessment of their own life conditions' quality (Seligson et al., 2003).

Importantly, the previous research has mainly focused on the individual level of well-being, ignoring issues at broader social levels. In order to overcome these limitations, the need for more comprehensive approaches to SWB has been emphasized (Ryan and Deci, 2001; Gallagher et al., 2009; Serban-Oprescu et al., 2019). In addition, different measures have been proposed, aiming to include additional complementary measures to capture the social aspects of SWB (Cummins, 2014). For example, additions of community- (Forjaz et al., 2010; Kim and Lee, 2013) and national-level (Morrison et al., 2011) SWB have been proposed to better account for the complex nature of well-being. According to the social–ecological approach (Bronfenbrenner, 1992), SWB at the individual, community, and national levels can be interpreted as the result of the interaction between individual meso- and macro-systems. Consequently, interactions with the place (i.e., community/neighborhood) and the country of residence may influence the individual SWB and vice versa.

While the individual SWB corresponds to the first level of deconstruction of life as a whole (Diener, 1994), emphasizing the meaning and self-realization when a person is fully functioning (Renn et al., 2008), the community SWB and national SWB included new relevant elements. On the one hand, the community SWB underscores the satisfaction with the local place of residence, including its broad range of economic, social, environmental, cultural, and governance conditions. On the other hand, the national SWB responds to a distal level of deconstruction, considering different societal conditions that may affect our lives (Morrison et al., 2011). Those recent constructs enable researchers to holistically understand the impact of community and country on individuals' SWB (Forjaz et al., 2010; Dronavalli and Thompson, 2015; McCrea et al., 2016; Atkinson et al., 2020).

### The Present Study

Different studies have analyzed the factor structures of different SWB scales from multiple theoretical perspectives in diverse contexts, populations, and languages, mainly using correlated-factor or higher-order models (i.e., Arthaud-Day et al., 2005; Tian et al., 2015; Nima et al., 2020). However, these models are limited in exploring complex constructs, such as SWB, presenting only a unidimensional or a multidimensional structure. In recent years, the development of the bifactors models has afforded analyses in which a group of items and their correlations are explained by a general factor that includes the shared variance of all or by a group of factors where the variance is partitioned (Rodriguez et al., 2016). Bifactor models are mainly used in psychopathology (e.g., Hammer and Toland, 2017; Zanon et al., 2020) but scarcer in the field of SWB (cf. Chen et al., 2006, 2013; Jovanović, 2015). To our knowledge, research has not tested the different levels of deconstruction of the SWB construct in a single model. Equally important is to identify different sources of variance of SWB.

**Figure 1:**
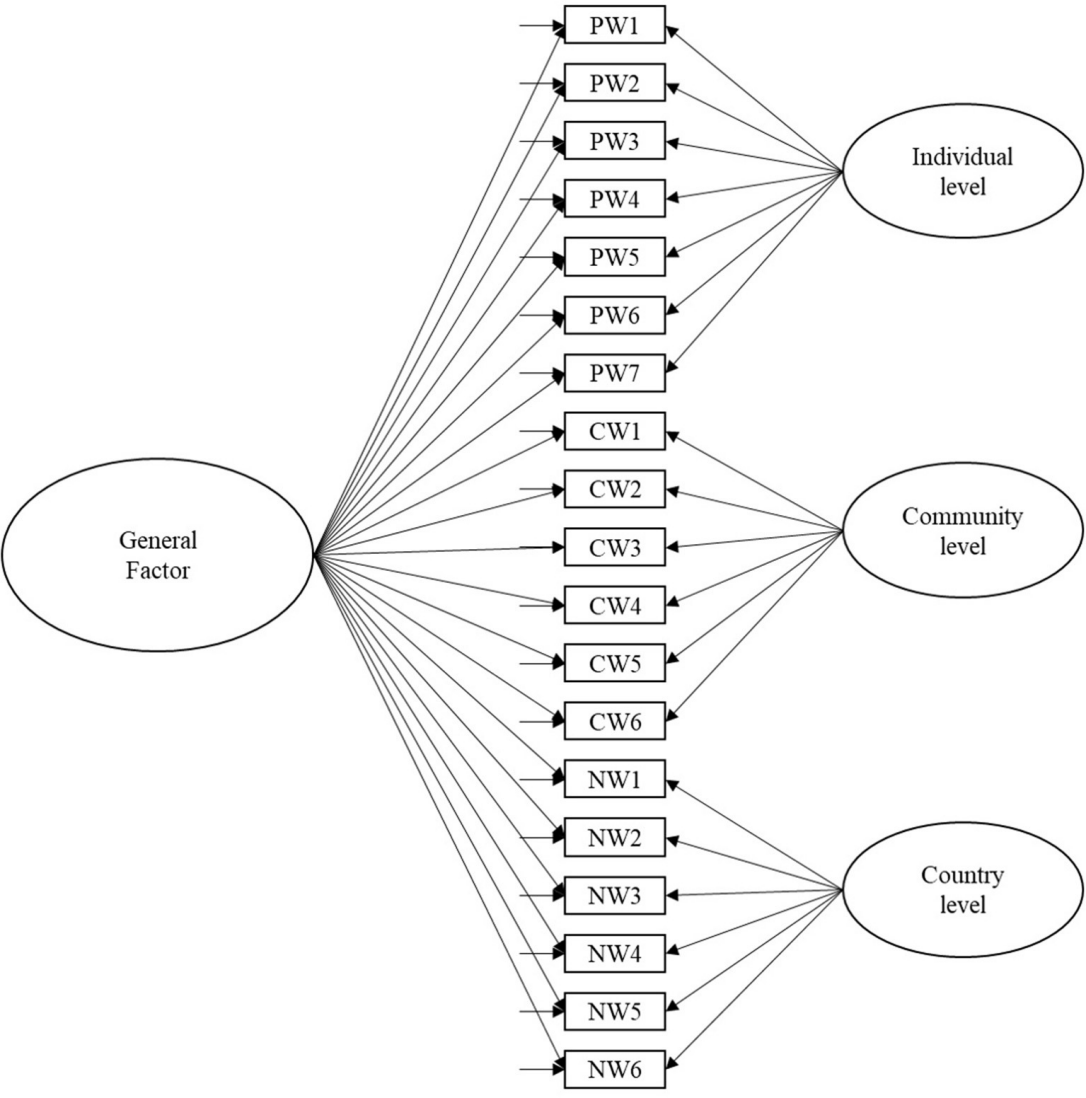
The conceptual bifactor model of subjective well-being.

To address these gaps, the present study examined the dimensionality and the reliability of a single measure of SWB, which contain individual, community, and national levels across three Latin-American countries' samples. In addition, the study examined whether the specific factors were associated only with the general measure of SWB rather than the particular factor. To do this, both general and specific factors were estimated simultaneously in the bifactor models. The main strength of the bifactor models is that they estimate the relation between latent variables, and they allow to measure a single common latent factor and control the variance that arises due to additional common factors (Reise et al., 2010). Figure 1 displays the conceptual model of the general measure of SWB.

## Methods

### Participants

The present study included a convenience sample of 2,616 adults from three main cities of Argentina, Chile, and Venezuela. The Argentinean sample (38.1%) consisted of 998 participants (46.2% females) from Buenos Aires, whose age ranged from 18 to 86 years (*M* = 34.72; *SD* = 13.26). About 58.2% of the sample completed their secondary education, while the remaining 41.8% indicated having completed or incomplete higher education. In terms of socio-economic background, participants were close to the middle-point of a scale measuring subjective socioeconomic status, ranging from 1 (lower status) to 5 (higher status) (*M* = 2.85, *SD* = 0.7, 62.6% in the midpoint).

The Chilean sample (38.2%) included 1,000 participants (55% females) from Santiago, with an age range of 18–85 years (*M* = 37.3; *SD* = 15.57). About 55.3% of the sample completed secondary education, while the remaining 44.7% had completed or incomplete higher education. In terms of socioeconomic background, the participants were close to the middle point of a scale, measuring subjective socioeconomic status ranging from 1 to 5 (*M* = 2.85, *SD* = 0.9, 52.5% in the midpoint).

Finally, the Venezuelan sample consisted of 618 participants (60.2% females) from Maracay, and their ages ranged from 18 to 89 years old (*M* = 43.46; *SD* = 15.97). About 58.5% of the sample had completed secondary education, while 41.5% reported an incomplete or completed higher education level. In terms of socioeconomic background, the participants were close to the middle-point of a scale, measuring subjective socioeconomic status, ranging from 1 to 5 (*M* = 2.92, *SD* = 1.58, 37.2% in the midpoint).

### Variables and Instruments

#### Personal Well-Being Index for Adults (PWI-A)

This scale was developed by the International Wellbeing Group (2013) by drawing on the Comprehensive Quality of Life Scale ComQoL (Cummins, 1997). It measures SWB in seven life domains: standard of living, personal health, achievement in life, personal relationships, personal safety, community connectedness, and future security. Lau et al. (2005) argued out that the scale captures the first-level deconstruction of life as a whole satisfaction and is broad enough to apply to several adult populations. It was adapted and validated in Chile by Oyanedel et al. (2015) with good reliability indicators (*N* = 400; α = 0.84). The reliability for the samples in the current study was acceptable as well (Argentina: α = 0.89; Chile: α = 0.82; Venezuela: α = 0.80).

#### Community Well-Being Index (CWI)

It is an adaptation of the *National Well-being Index* (NWI) as a community-level measure of SWB. Developed by Forjaz et al. (2010), originally in Spanish, the scale contains six items, assessing satisfaction with the community's living conditions: economic situation, state of the environment, social conditions, community government, security, and business. The internal consistency for this scale for the current study was 0.89 for Chile and 0.90 for Argentina and Venezuela.

#### National Well-Being Index (NWI)

It is a six-item scale that measures satisfaction with living conditions in a country. Developed by Cummins et al. (2003) and translated to Spanish by Rodriguez-Blazquez et al. (2010), it taps into satisfaction with the country's economic situation, state of the environment, social conditions, community government, security, and business. We observed good reliability coefficients in all samples, ranging from 0.87 for Chile and 0.94 for Venezuela.

For the three instruments, each item is scored on an 11-point Likert scale (from 0 = “Not satisfied at all” to 10 = “Completely satisfied”).

### Procedure

We collected the data in the three countries between 2018 and 2019. We reached the participants, through interviewers previously trained, at their households. In each main city, we followed a multistage random sampling procedure to recruit participants. First, we randomly selected blocks in each city. Second, within every block, we randomly selected at least 50% of houses. To reduce potential selection biases, we did not select the houses next to each other in the same block, selecting only the odd-numbered houses, starting from the northeast corner of the block. Finally, in every selected house, the interviewer asked the householder to participate in the study. If he/she was not present at the time of the visit, we revisited the householder at another time that he voluntarily indicated.

Prior to data collection, we had trained the interviewers through classroom training, where we introduced them to the survey, methods for selecting houses for each block, and for registration of the surveys conducted. As a control method, we voluntarily asked for the telephone number of the head of the household, and then we later selected a random subsample of the participants and contacted them to confirm the application of the survey and some sociodemographic data.

Our intention was not to collect representative samples from each country; consequently, convenience sampling was conducted in the three countries to investigate SWB at three levels, that is, personal, community, and country levels.

### Analytic Strategy

The data were analyzed, using Mplus 7.3 (Muthén and Muthén, 2012). Missing values were treated as pairwise missing, assuming missing is MAR or MCAR (Lei and Shiverdecker, 2019).

To evaluate the best factor structure for a general measure of SWB, we tested four different models *via* confirmatory factor analysis (CFA) for the total sample. First, in a *unidimensional* model, all 19 items were loaded to a single latent factor. Second, a *three-factor* model was tested in which each item was set to a load in its specific first-order factor (PWI-A, CWI, NWI), and correlations between these factors were tested. Third, a *second-order* model was examined. In this model, a higher-order structure was set to predict the specific factors (or measures). Finally, a *bifactor* model specified that each item would load into a general common factor (SWB) as well as its specific factors (PWI-A, CWI, NWI). The general factor arguably represented a broad central construct, and group factors represented SWB's particular subdomains (Rodriguez et al., 2016). All factors were set orthogonal to each other, meaning they were not allowed to correlate (Hammer and Toland, 2017).

The models were estimated, using the robust Maximum Likelihood (MLR) estimation method. Goodness of a fit was calculated, including Satorra-Bentler chi-square (SBχ^2^), comparative fit index (CFI), root mean square error of approximation (RMSEA), and standardized root mean square residual (SRMR). A CFI ≥ 0.95 and RMSEA and SRMR ≤ 0.05 were considered a very good fit (see Hu and Bentler, 1999; Batista-Foguet and Coenders, 2012; Arbuckle, 2014). Only the models that achieved an adequate fit were improved *via* modification indices.

We tested the measurement invariance of the structural model that showed a good fit between the three countries at three levels: *configural* (same items loading onto the same latent variables), *metric* (factor loading constrained), and *scalar* (factor loadings, intercepts, and factor means constrained) (Meredith, 1993). As a sign of invariance, a non-significant change in χ^2^ was used (Millsap and Olivera-Aguilar, 2012), or a change in the CFI (ΔCFI) < 0.010 (Cheung and Rensvold, 2002; Chen, 2007; Millsap, 2012) was supplemented by ΔRMSEA < 0.015 (Putnick and Bornstein, 2016). This is an incremental measure; more and more constraints are added to the model to test to what level they are comparable to each other.

If PWI-A, CWI, and NWI conformed to a bifactor structure, this would indicate that SWB might be evaluated as both unidimensional and multidimensional measurement model (Chen et al., 2006). Hence, it is essential to know how much composite items' variances are attributed to the general factor or the specific factors and how much their internal consistency scores are inflated for that reason (Zanon et al., 2020). Ancillary bifactor indices are required to estimate the model-based reliability of general or subscale scores and the dimensionality of the instrument (Rodriguez et al., 2016). *Coefficient omega* (ω) estimates the proportion of total score variance attributable to all sources of common variance. *Coefficient omega hierarchical* (ω_H_) estimates the proportion of total score variance attributed to a single general factor, after accounting for specific factors as measurement errors. *Coefficient omega hierarchical subscale* (ω_S_) is an extension of the previous one (i.e., ω_H_), and it reflects the proportion of variance in the composite specific factor, after controlling for the variance due to the general factor. A high ω_H_ (>0.75) would indicate a presence of a single general factor, supporting the use of the raw total score. At the same time, a high ω_H_ reflects the predominance of the specific factor as a source of variance. *Proportion of reliable variance* (PRV) to general and group factors refers to the reliable variance accounted for by that factor (Hammer and Toland, 2017). Finally, *explained common variance* (ECV) is an index of unidimensionality, and it indicates the proportion of common variance across items explained by the general factor (Zanon et al., 2020).

### Ethics Statement

This study was carried out following the recommendations of the National Agency of Science and Technology of Chile with written informed consent from all the participants. The protocol was approved by the ethics committee of University Andres Bello.

## Results

Table 1 presents descriptive statistics and Cronbach's alpha coefficients for all measures for each country sample. All internal consistency estimates (α) were higher than 0.80 in all cases. Even though it was not possible to meaningfully compare scores between the countries, the results showed that Chile presented the highest global scores for all scales. At the same time, Venezuela showed lower scores for CWI and NWI and Argentina in PWI-A. Also, skewness and kurtosis of all scales and their items did not show problematic values for normality, according to Kline (2015).

**Table 1:** Descriptive statistics and differences between countries.

	**Cronbach's** **α**			***M*** **(*****SD*****)**		
	**Argentina**	**Chile**	**Venezuela**	**Argentina**	**Chile**	**Venezuela**
PWI–A	0.899	0.820	0.807	6.76 (1.42)	7.47 (1.54)	6.95 (1.90)
CWI	0.895	0.891	0.898	5.11 (1.55)	5.66 (1.91)	3.81 (2.46)
NWI	0.894	0.869	0.942	4.59 (1.50)	4.99 (1.81)	1.88 (2.40)

*PWI-A, personal well-being index for adults. CWI, community well-being index. NWI, national well-being index. All differences between countries were statistically significant (p < 0.001)*.

In order to test the structure of the different measures of SWB, including the three scales, a series of CFAs were tested, considering four competing measurement models: unidimensional, three-factor, second-order, and bifactor models. As shown in Table 2, the unidimensional, the three-correlated factors, and the second-order models yielded poor fitting for the total sample. On the other hand, the bifactor model showed an acceptable fit to the data. An evaluation of the modification indexes suggested that its fit should improve by releasing the correlation between some items: For PWI-A, item 5 “*How safe you feel*” and item 7 “*Future security*,” and item 7 with item 6 “*Feeling part of the community*”; and for NWI: item 3 “*National social conditions*” and item 2 “*State of the environment of the country*.” The refined bifactor model resulted in a better fit to data for the total sample. These results may suggest that the bifactor model best represents the structure of a general measure of SWB.

**Table 2:** Summary of fit indices for structural models of SWB for the total sample.

**Model**	**SB _χ^2^_**	**df**	**CFI**	**RMSEA [90% CI]**	**SRMR**
Single-factor model	7299.332^***^	152	0.606	0.134 [0.131–0.137]	0.141
Three-factor model	1998.090^***^	149	0.898	0.069 [0.066–0.072]	0.058
Second-order factor model	1998.096^***^	149	0.898	0.069 [0.066–0.072]	0.058
Bifactor model	1470.486^***^	133	0.926	0.062 [0.059–0.065]	0.047
Bifactor model + CE	801.937^***^	130	0.963	0.045 [0.042–0.048]	0.031

*** *p < 0.001. CE, correlated errors*.

Consequently, we examined the measurement invariance of the bifactor model across the countries, using hierarchically and increasingly restrictive models: configural, metric, and scalar (Table 3). Evidence for measurement invariance is necessary before scores as single observations or higher construct can be meaningfully compared across groups. Configural invariance was established for the bifactor model structure (CFI = 0.936, RMSEA = 0.055). Then, by restricting the factor loadings to be equal across the countries, we tested the metric model. Although it presented an acceptable fit, the SBχ^2^ difference between the metric and the configural model was significant (*p* < 0.001), and ΔCFI was >0.010, both suggesting that metric invariance was not supported. These results show that factor loadings are not equivalent across the countries.

**Table 3:** Multigroup confirmatory factor analysis of the bifactor model of SWB.

		**SB_χ^2^_ (df)**	**CFI**	**RMSEA**	**Δ SB_χ^2^_ (df)**	**ΔCFI**	**ΔRMSEA**	**p (SB_χ^2^_)**
1	Configural	1546.897^***^ (390)	0.936	0.055	–	–	–	–
2	Metric	1906.216^***^ (458)	0.919	0.058	359.319 (68)	−0.017	0.003	0.000
3	Scalar	2287.367^***^ (488)	0.900	0.063	381.151 (30)	−0.019	0.005	0.000

*** *p < 0.001*.

Considering these results, the bifactor model was calculated for each country, as well as its ancillary indices. Table 4 presents standardized factor loadings, sources of variance in SWB, and reliability estimates for all general and three specific factors. For the total sample bifactor model, CWI and NWI items had strong loadings (>0.594) on the general factor, while PWI-A items had lower factor loadings, ranging from 0.187 to 0.429. Considering the model-based reliability, omega coefficients showed that 94.9% of the total score variance was due to all common factors, general and specifics, and 83.2–93.8% of the subscale score variance was due to general and that specific factor. Omega hierarchical coefficients showed a predominance of the general factor over the specific factors, where 72.9% of the variance could be attributed exclusively to the general factor. On the other hand, the analysis of omega hierarchical subscale coefficients indicated that PWI-A also exhibited a high value (ω_S_ = 0.654), unlike CWI (ω_S_ = 0.240) and NWI (ω_S_ = 0.388). PRV inspection showed the same result: PWI-A can be analyzed both as part of the general factor (PRV = 76.9%) and as a specific factor (PRV = 78.7%). Analysis for the model-based dimensionality—ECV—did not reach the benchmark to consider SWB as essentially unidimensional (ECV = 55.4%). These results support the idea that, for the total sample, the SWB construct can be treated in a unidimensional and a multidimensional way since neither of them predominates.

**Table 4:** Standardized factor loadings, construct reliability, and sources of variance for the bifactor model of SWB.

	**Total sample**				**Argentina**				**Chile**				**Venezuela**			
	**Gen**	**PWI-A**	**CWI**	**NWI**	**Gen**	**PWI-A**	**CWI**	**NWI**	**Gen**	**PWI-A**	**CWI**	**NWI**	**Gen**	**PWI-A**	**CWI**	**NWI**
PW1	0.284^*^	0.631^*^			0.317^*^	0.595^*^			0.316^*^	0.683^*^			0.187^*^	0.602^*^		
PW2	0.143^*^	0.583^*^			0.359^*^	0.639^*^			0.196^*^	0.548^*^			0.150^*^	0.488^*^		
PW3	0.260^*^	0.747^*^			0.360^*^	0.765^*^			0.235^*^	0.712^*^			0.313^*^	0.715^*^		
PW4	0.187^*^	0.673^*^			0.389^*^	0.731^*^			0.197^*^	0.611^*^			0.183^*^	0.589^*^		
PW5	0.429^*^	0.475^*^			0.481^*^	0.514^*^			0.322^*^	0.445^*^			0.494^*^	0.417^*^		
PW6	0.294^*^	0.481^*^			0.483^*^	0.545^*^			0.303^*^	0.373^*^			0.415^*^	0.393^*^		
PW7	0.424^*^	0.435^*^			0.559^*^	0.445^*^			0.325^*^	0.377^*^			0.498^*^	0.348^*^		
CW1	0.661^*^		0.445^*^		0.481^*^		0.635^*^		0.686^*^		0.415^*^		0.577^*^		0.450^*^	
CW2	0.603^*^		0.612^*^		0.529^*^		0.664^*^		0.658^*^		0.566^*^		0.600^*^		0.626^*^	
CW3	0.594^*^		0.693^*^		0.373^*^		0.801^*^		0.682^*^		0.533^*^		0.634^*^		0.678^*^	
CW4	0.691^*^		0.361^*^		0.269^*^		0.762^*^		0.671^*^		0.247		0.746^*^		0.260^*^	
CW5	0.784^*^		0.137^*^		0.405^*^		0.547^*^		0.646^*^		0.118		0.784^*^		0.074	
CW6	0.746^*^		0.228^*^		0.447^*^		0.452^*^		0.767^*^		0.196^*^		0.771^*^		0.085	
NW1	0.678^*^			0.568^*^	0.469^*^			0.683^*^	0.527^*^			0.559^*^	0.574^*^			0.673^*^
NW2	0.614^*^			0.488^*^	0.697^*^			0.379^*^	0.483^*^			0.591^*^	0.597^*^			0.480^*^
NW3	0.608^*^			0.638^*^	0.531^*^			0.661^*^	0.450^*^			0.658^*^	0.583^*^			0.702^*^
NW4	0.595^*^			0.651^*^	0.408^*^			0.748^*^	0.459^*^			0.595^*^	0.545^*^			0.747^*^
NW5	0.692^*^			0.459^*^	0.440^*^			0.588^*^	0.503^*^			0.391^*^	0.645^*^			0.520^*^
NW6	0.681^*^			0.446^*^	0.513^*^			0.407^*^	0.583^*^			0.369^*^	0.617^*^			0.542^*^
ω =	0.949	0.832	0.920	0.938	0.938	0.896	0.901	0.905	0.921	0.793	0.901	0.872	0.944	0.803	0.915	0.942
ω_H_ =	0.729	0.091	0.043	0.085	0.578	0.143	0.117	0.101	0.696	0.103	0.039	0.084	0.734	0.082	0.039.	0.089
ω_S_ =	–	0.654	0.240	0.388	–	0.597	0.641	0.518	–	0.625	0.181	0.458	–	0.551	0.199	0.482
PRV (%)	76.9	78.7	26.1	41.3	61.5	66.7	71.1	57.3	75.5	78.9	20.1	52.5	77.8	68.7	21.7	51.2
ECV (%)	55.4	17.1	10.6	17.8	34.7	25.9	22.4	20.0	53.2	19.8	9.9	18.1	55.1	16.2	11.4	18.7

*PWI-A, personal well-being index for adults; CWI, community well-being index; NWI, national well-being index*.

* *Significant standardized factor loadings (p < 0.05). ω, omega coefficient; ω_h_, omega hierarchical coefficient; ω_s_, omega subscale coefficient; PRV, proportion of reliable variance; ECV, proportion of explained common variance*.

The bifactor model in Argentina's sample showed a good fit to the data (SBχ^2^ = 617.163, df = 130, *p* < 0.001, CFI = 0.931, RMSEA = 0.061, 90% C.I. [0.057, 0.066], SRMR = 0.047). The factor loadings of the model suggest that they mainly loaded higher toward the specific factors than the general factor: six out of seven items of PWI-A, all items of CWI, and four out of six items of NWI. The overall model reliability was high (ω = 0.938), which means that 93.8% of total score variance could be attributed to all common factors. Nevertheless, the omega hierarchical for the total score was lower than the total sample (ω_H_ = 0.578), indicating that a less variance could be attributed to the general factor after controlling for all specific factors. Similar values were obtained for the omega subscale coefficients but higher than the previous one, except for NWI (ω_S_ = 0.518). When we added the PRV to this analysis, only 61.5% accounted for the general factor but higher values to specific factors of PWI-A (PRV = 66.7%) and CWI (PRV = 71.1%). These results may suggest that the SWB construct for Argentina is in a gray area between a broader general factor and narrower specific factors. When the ECV was considered for dimensionality, all values were below 0.70, supporting this conclusion.

In the case of Chile as well, the bifactor model showed an acceptable fit to the data (SBχ^2^ = 577.645, df = 130, *p* < 0.001, CFI = 0.923, RMSEA = 0.059, 90% C.I. [0.054, 0.064], SRMR = 0.042), but it presented different patterns in terms of factor loadings. In this case, PWI-A and NWI (four out of six items) tended to load higher in their specific factors than the general factor; however, this was not the case for CWI whose factor loadings were high, ranging between 0.646 and 0.767. The omega reliability coefficients were adequate for both the general (ω = 0.921) and specific factors (0.793 to 0.901). The omega hierarchical coefficient showed that 69.6% of the total score variance could be attributed to the general factor after controlling for all specific factors. In contrast, the omega subscale coefficients indicated less proportion of subscale score variance, explained by factors controlling the general factor (PWI-A: ω_S_ = 0.625, CWI: ω_S_ = 0.181, NWI: ω_S_ = 0.458). Considering the PRV indicators, we could observe that both the proportions of reliable variance of the general factor (PRV = 75.5%) and the PWI-A (78.9%) were higher than the cutoff point of 0.75, indicating that this factor could be explained in both ways. Also, dimensionality analyses showed that ECV only reached 53.2% for the general factor, supporting both unidimensionality and multidimensionality of SWB.

Finally, in the Venezuela's sample, we also observed a good fit of the bifactor model to the data (SBχ^2^ = 340.068, df = 130, *p* < 0.001, CFI = 0.958, RMSEA = 0.051, 90% C.I. [0.045, 0.058], SRMR = 0.036). Regarding the factor loadings for the model, four out of seven items of PWI-A and three out of six items of NWI loaded higher in their specific factors. Reliability measures by the omega coefficient were good for general (ω = 0.944) and specific factors (0.942 to 0.803). The omega hierarchical coefficient showed a clear predominance of the general factor (ω_H_ = 0.734) over the specific factors according to the omega subscale (PWI-A: ω_S_ = 0_.5_51, CWI: ω_S_ = 0.199, NWI: ω_S_ = 0.482). This means that 73.4% of the total score variance could be attributed to the general factor after accounting for all specific factors. However, PRV showed 77.8% of reliable variance due to the general factor independent of the specific factors, and 68.7% due to PWI-A independent of the general factor. These results indicate a predominantly unidimensional structure of SWB. ECV showed that 55.1% of the common variance across items was explained by the general factor. Although these values do not necessarily imply a unidimensionality of SWB, they are supportive of this conclusion.

## Discussion

The main objective of this study was to evaluate the SWB structural model, considering the individual (PWI-A), community (CWI), and national (NWI) levels as factors, as a more comprehensive measure. Overall, the results support that SWB is better analyzed as a complex construct, considering different sources of information at different levels (Bronfenbrenner, 1992; Gallagher et al., 2009; Jovanović, 2015). Four models were tested to know the relationship between the three scales or factors and determine which one best summarized its factor structure. The single-factor, three-factor, or second-order models showed a poor fit to the data. However, the bifactor model showed a good fit, which was further improved after including some error covariances. These modifications were theory driven (e.g., Oyanedel et al., 2015) and supported by the data through modification indices, guided by the authors' criteria. For instance, PWI-A item 5 “*How safe you feel*” (“*Cuánseguro/a te*sientes”) and PWI-A item 7 “*Future security*” (“*Tu seguridadfutura*”) have a similar translation in the Spanish version. It makes sense that the two items were closely related.

We examined measurement invariance only for the bifactor model across the three countries because it was the only model that showed a good fit to the data. This model showed an acceptable fit for the configural invariance across the countries, but it did not provide enough evidence for metric or scalar invariance. This implies that the factor structures may be equivalent, but not their factor loadings or their latent means; thus, no meaningful comparisons across the countries could be made. These findings are supported by previous cross-cultural studies that found that PWI is not invariant across countries, only reaching partial metric/scalar invariance (Zemojtel-Piotrowska et al., 2016) or had been modified from its original structure. Considering cross-cultural differences (e.g., how items are understood) is pivotal when analyzing these results (Jovanović et al., 2018).

The above suggests that there are differences in the notion of SWB or its components, which highlights the importance of country-specific models. The results for bifactor models across countries, in general, showed that SWB was in a gray area between unidimensionality and multidimensionality but with different nuances. In Argentina's results, most of the factor loadings were observed on specific factors. However, more than half of the reliable variance was accounted for by the general factor and the specific factors. Chile's case was different: All PWI-A items presented higher factor loadings on the specific factor—most of the NWI and none of the CWI. The proportion of reliable variance was high in the general factor and PWI-A specific factors. ECV indicated that the proportion of explained common variance could suggest a mostly unidimensional structure. In Venezuela's case, most of the factor loadings were on the general factor, but the proportion of reliable variance was high in general and PWI-A specific factors. These results may suggest a structure of personal well-being that can be attributed to both the general factor and its specific factors.

Cross-cultural differences can be explained basing on the ecological model (Bronfenbrenner, 1992): As far as the personal well-being is the closest level of well-being, it immediately affects the individual's perception of his/her own life. The perception of the well-being of the community and the country, being a mesosystem and a macro-system, respectively, indirectly affects the SWB. It is important to recall that, at these levels, the source of well-being is not placed in an individual.

In comparison with individual SWB, the national level SWB has received less attention, despite its relevance in modern countries (Eker and Ilmola-Sheppard, 2020). While SWB research has focused on its effects on individual- and societal-level explanations, a mixed approach, linking both perspectives—a subjective approach to institutional assessment and a societal approach to SWB—has not yet been fully developed. An essential part of the public policies that seek to reduce inequality and promote mental health is to legislate for people's well-being (Jenkins, 2003). The current challenge modern nations face is to integrate the dimension of people's subjectivity as a transcendental axis in the political scenario (Kroll and Delhey, 2013). This is because it has become clear that those preferably external indicators—mainly economic—on which national and global progress has traditionally been based have important limitations on knowing how satisfied people are with their lives (Unanue, 2017). Therefore, the current study explores to rethink human development, considering integral SWB, which assumes both the dimension of personal SWB—people's satisfaction with their own lives—and that of SWB with society—an evaluation made by the people of the society in which they live.

At the social level, the impact of Latin American countries' different sociopolitical realities in recent years has had a differential impact on each nation and individual. To this end, one of the strengths of the current study is that it considers samples from three different countries. Latin America is experiencing a period of discontent with democracy since the past years, which is reflected in democratic disaffection, a lack of trust in institutions, and questioning of forms of government, among others (Sanahuja, 2019). In fact, this dissatisfaction can be observed on each of the scales' scores across the three countries—consistently, the lowest scores were observed at the national level, followed by the community level—finally, the personal level with values above the theoretical mean of the scale. In addition, the lowest scores were observed in Venezuela—the country that for several years has been experiencing a severe social, economic, and political crisis, resulting in generalized unrest and subsequent massive migration, mainly to other Latin American countries, a phenomenon associated with a feeling of constant lack of protection (Gandini et al., 2020).

Additionally, the current research also highlights the necessity of considering the SWB from a multilevel approach. To be able to precisely understand and measure the complexity of the SWB is determinant for future intervention proposals and, in turn, to reach the global agenda well-being goals.

### Limitations and Future Directions

The study comes with a few limitations. First, the use of non-representative samples from each country makes it unlikely that results can be generalized; we tried to obtain a heterogeneous and random sample, but, since people choose whether to participate, this could imply analyzing the results with caution. However, it does provide indicators of SWB at different levels. Second, the non-invariance between countries makes it irrelevant to conduct group comparisons. Future studies should carry out restrictions and semi-partial models to analyze where the differences in SWB measures are. Third, the bifactor analysis shows that there are indicators of well-being that are common and others that are specific; however, this type of analysis allowed us to learn more about which ones contribute specifically as domains and which ones contribute to a general construct for each country, developing specific models for each sample. Future research should investigate which factors are common and which are not in order to discern between the global part of well-being and the specific parts. It is also necessary to include some control variables as socioeconomic status or gender in the structural models, as these have been shown to be relevant aspects to SWB.

## Data Availability Statement

The datasets generated during and/or analyzed during the current study are not publicly available because this is an ongoing investigation that ends in 2022 and not all available analyses have been completed. However, datasets may be available from the corresponding author on reasonable request.

## Ethics Statement

The studies involving human participants were reviewed and approved by University Andrés Bello. The patients/participants provided their written informed consent to participate in this study.

## Author Contributions

JT-V: led the research, data analyses, results, discussion, and drafted the manuscript. JJ-B: co-led the research, the data analyses, the interpretation of the data, and drafted and commented on the manuscript. JO: data collection, review of the literature, and the final version of the manuscript. MS: review of the literature and references. All authors contributed to the article and approved the submitted version.

## Conflict of Interest

The authors declare that the research was conducted in the absence of any commercial or financial relationships that could be construed as a potential conflict of interest.
