# Supplementary material for: A Bifactor Model of Subjective Well-Being at Personal, Community, and Country Levels: A Case With Three Latin-American Countries
Source: Front Psychol. 2021 Jun 3;12:641641. doi: 10.3389/fpsyg.2021.641641 (PMC8209295; doi:10.3389/fpsyg.2021.641641)
Supplement: Supplementary file 1 [file Table_1.pdf]

Supplementary Material

Personal Well-being Index for adults (PWI-A). The International Wellbeing Group (2013) and Oyanedel et al. (2015) version.

	English wording	Spanish wording
	How satisfied are you with…?	¿Qué tan satisfecho/a estás con…?
1	your standard of living?	Su nivel de vida
2	your health?	Su estado de salud general
3	what you are achieving in life?	Los logros que está alcanzando en su vida
4	your personal relationships?	Las relaciones personales
5	how safe you feel?	Lo seguro y protegido que usted se siente
6	feeling part of your community?	Su sentimiento de formar parte de la comunidad en que vive
7	your future security?	Su seguridad y protección futura

Community Well-being Index (CWI). Forjaz et al. (2010).

	English wording	Spanish wording
	How satisfied are you with the following things in your community/town/city…?	¿Qué tan satisfecho/a estás con las siguientes cosas en su comuna/comunidad…?
1	The economic situation of your town or city	La situación económica de su comuna/comunidad
2	The state of environment	El estado del medioambiente de su comuna/comunidad
3	The social conditions of your town or city	Las condiciones sociales de su comuna/comunidad
4	The government	El gobierno comunal
5	Business activity	Las posibilidades que tienen las empresas y las personas de hacer negocios en su comuna/comunidad
6	Security	La seguridad en su comuna

National Well-being Index (NWI). Cummins et al. (2003) and Rodriguez-Blazquez et al. (2011) version.

	English wording	Spanish wording
	How satisfied are you with the following things in your country…?	¿Qué tan satisfecho/a estás con las siguientes cosas en su país…?
1	The economic situation of your country	La situación económica de su comuna/comunidad
2	The state of environment	El estado del medioambiente de su comuna/comunidad
3	The social conditions of your country	Las condiciones sociales de su comuna/comunidad
4	The government	El gobierno comunal
5	Business activity	Las posibilidades que tienen las empresas y las personas de hacer negocios en su comuna/comunidad
6	Security	La seguridad en su comuna
